# Is gaming disorder related to psychological trauma? A scoping review

**DOI:** 10.1192/j.eurpsy.2024.666

**Published:** 2024-08-27

**Authors:** H. W. Fung, C. T. Y. Cheung, P. Lam, G. F. Yuan, M. Y. C. Wong, H. W.-H. Ling, S. K. K. Lam, A. K. C. Chau, V. W. P. Lee

**Affiliations:** ^1^Department of Social Work, Hong Kong Baptist University; ^2^Nethersole School of Nursing, Faculty of Medicine, The Chinese University of Hong Kong, Hong Kong, Hong Kong; ^3^Institute of Sociology, National Tsinghua University, Hsinchu, Taiwan, Province of China; ^4^South Carolina SmartState Center for Healthcare Quality, Arnold School of Public Health; ^5^Department of Health Promotion, Education, and Behavior, Arnold School of Public Health, University of South Carolina, Columbia, SC, United States; ^6^Department of Health and Physical Education, The Education University of Hong Kong; ^7^Department of Social Work and Social Administration, The University of Hong Kong; ^8^Institute of Health Equity, The Chinese University of Hong Kong, Hong Kong, Hong Kong

## Abstract

**Introduction:**

Gaming disorder has become a global concern and it could have a variety of health and social consequences. The trauma model has been applied to the understanding of different types of addictions as behavioral addictions can sometimes be conceptualized as self-soothing strategies to avoid trauma-related stressors or triggers. However, much less is known about the relationship between trauma exposure and gaming disorder.

**Objectives:**

To inform prevention and intervention strategies and to facilitate further research, we conducted the first scoping review to explore and summarize the literature on the relationship between trauma and gaming disorder.

**Methods:**

A systematic search was conducted on the Web of Science, Scopus and ProQuest. We looked for original studies published in English that included a measure of trauma exposure and a measure of gaming disorder symptoms, as well as quantitative data regarding the relationship between trauma exposure and gaming disorder.

**Results:**

The initial search generated 412 articles, of which 15 met the inclusion criteria. All of them were cross-sectional studies, recruiting participants from both clinical and non-clinical populations. Twelve of them (80%) reported significant correlations between trauma exposure and the severity of gaming disorder symptoms (r = 0.18 to 0.46, p < 0.010). Several potential mediators, including depressive symptoms and dissociative experiences, have been identified. One study found that parental monitoring moderated the relationship between trauma and gaming disorder symptoms. No studies reported the prevalence of trauma or trauma-related symptoms among people with gaming disorder.

**Conclusions:**

There is some evidence supporting the association between trauma and gaming disorder, at small to medium effect sizes. Future studies should investigate the mediators and moderators underlying the relationship between trauma and gaming disorder. The longitudinal relationship between trauma exposure and the development of gaming disorder should be clarified. A trauma-informed approach may be a helpful strategy to alleviate gaming disorder symptoms.

**Disclosure of Interest:**

None Declared

